# Basilar Artery Band Thrombus: Report of a Unique Case

**DOI:** 10.7759/cureus.68704

**Published:** 2024-09-05

**Authors:** Kazzara T Raeburn, Kathleen C Bubb, Yoko Tabira, Joe Iwanaga, R. Shane Tubbs

**Affiliations:** 1 Anatomical Sciences, St. George's University, St. George's, GRD; 2 Anatomy Division, Weill Cornell Medicine, New York, USA; 3 Department of Anatomy, Kurume University School of Medicine, Kurume, JPN; 4 Neurosurgery, Tulane University School of Medicine, New Orleans, USA; 5 Neurosurgery and Ochsner Neuroscience Institute, Ochsner Health System, New Orleans, USA

**Keywords:** basilar artery bands, plaque, stroke, thrombus, vertebrobasilar circulation

## Abstract

Basilar artery bands (BAB) are described in scant reports in the literature as shelves, bridges, septa, or webs within the lumen of the basilar artery. The anatomy and histology of these bands have only recently been fully explored and classified. Although there has been evidence of non-atherosclerotic calcification of these structures and vertebrobasilar atherosclerosis, previous studies have not demonstrated any plaque or thrombus formation on the basilar artery bands. Herein, we report the unique finding of a thrombus associated with a BAB. This case report warns interventionalists and neurosurgeons that there could be a thrombus around the BAB.

## Introduction

Basilar artery bands (BAB) are described in reports in the literature as shelves, bridges, septa, cords, fibers, pillars, or webs [1-6]. Unlike the more commonly seen basilar artery fenestrations (BAF), they are rarely discussed. A prevalence of between 0.6% on CT angiograms [4] and up to an average of 10% on cadaveric studies [2,6] has been established. The anatomy and histology of these intraluminal bands have only recently been fully explored and classified [2]. Intraluminal structures have been previously described in cerebral arterial and venous vessels, including the carotid artery, the anterior communicating artery, and the transverse and superior sagittal sinuses [3,5,7]. Associations have been made between cerebral infarcts and intra-arterial webs [8-12]. Although Liu et al. demonstrated plaque formation on basilar artery fenestrations, the association between basilar artery fenestration and stroke is unknown [13]. Previous studies have not shown any plaque or thrombus formation on BAB [2,4,6]. However, there has been evidence of non-atherosclerotic calcification of these structures [4] and vertebrobasilar (VB) atherosclerosis [2]. This paper discusses the unique finding of a thrombus associated with a BAB.

## Case presentation

**Figure 1:**
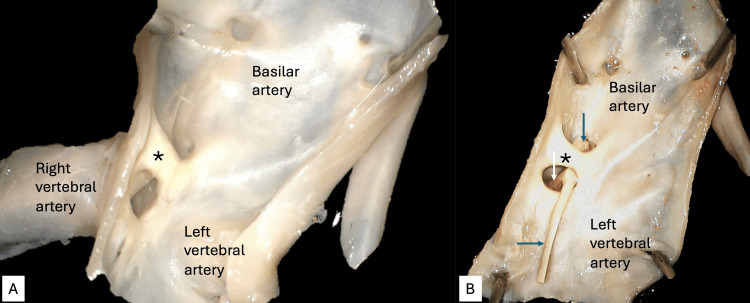
Basilar artery band A: Opened left vertebral artery noting the basilar artery band (*). B: Opened left vertebral artery noting the basilar artery band (*) with thrombus (arrows).

During the routine dissection of a 72-year-old at-death male cadaver, an intra-arterial band was identified at the vertebrobasilar junction (Figure 1).

**Figure 2:**
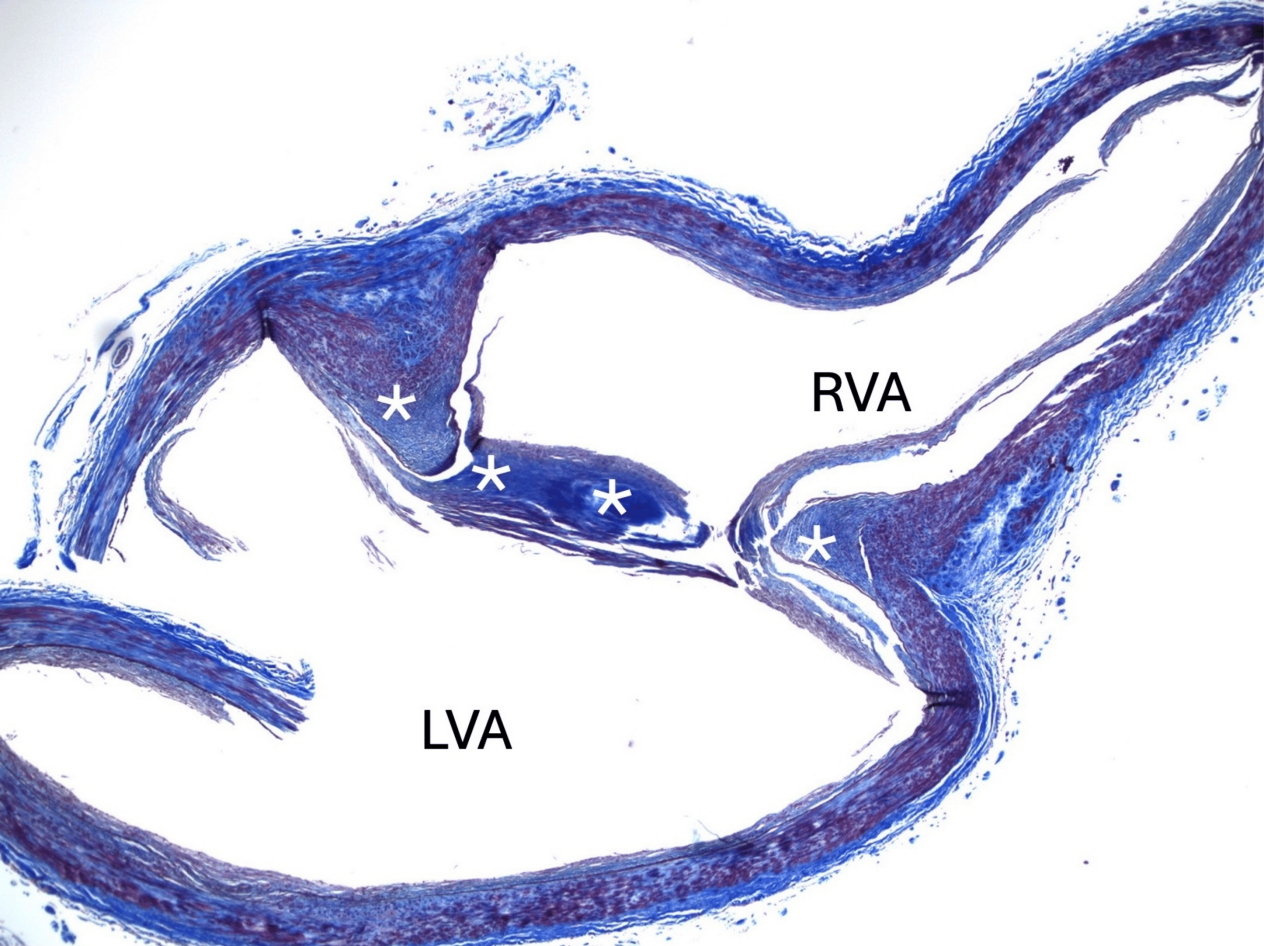
Histology study Histological image with Masson Trichrome staining through the basilar artery band (*) between the terminal left (LVA) and right (RVA) vertebral arteries, i.e., proximal basilar artery.

Specifically, the BAB was found at the junction of the left and right vertebral arteries. The band traveled, more or less, from the anterior wall of the vessel to its posterior wall and, using the classification of Glennon et al. [2], was a type 1 band. The length of the band was 0.22 mm, and its height was 0.12 mm. Additionally, related to the band was a Y-shaped thrombus with arms in the left and right vertebral arteries and a body extending to the left of the BAB into the basilar artery. No other thrombi were noted in the vertebrobasilar system or anterior circulation. Histologically, the BAB was primarily an extension of the endothelium with a base at each end of its attachment (Figure 2).

No gross findings of cerebral infarction were noted in the distribution of the posterior circulation. The cause of death of this cadaver donor was listed as cardiac arrest. 

## Discussion

To our knowledge, a case of a thrombus related to a BAB has yet to be reported in the literature. Even in case it existed, the thrombus might have been removed before detailed investigation. Although the exact embryologic origins of the BAB remain undetermined, Small et al. have proposed that these bands can be considered “aberrant BAF.” Tubbs et al. posited that these bands represent a “forme fruste” of BAF fenestrations due to incomplete fusion of the fetal longitudinal vessels that form the basilar artery [6]. Stehbens has suggested that these bands arise from the incomplete fusion of two neighboring arteries involving their primitive capillary networks. 

Glennon et al. categorized the BAB into type 1 (vertical) and type 2 (horizontal) bands [2]. The prevalence of the vertebral bands was 7.5% (6 of 80 specimens), while the prevalence of the horizontal bands was 2.5% (2 of 80 specimens) [2]. The vertical bands in the sagittal plane may share the exact embryological origins of BAF [2]. However, the horizontal bands may be entirely unrelated [2]. Small et al. described the BAB as linear or nodular, with 57% of the bands being elongated linear structures and 43% having bulges and protuberances [4]. Previous studies found BAB to be predominantly located in the inferior part of the basilar artery near the VB junction [1,2,4,6], with a prevalence of 66.7% [4] to 100% [2, 6]. BAB should be considered when treating basilar tip aneurysm with endovascular coils [14] or when placing stents into the basilar artery [15].

Histologically, BABs are extensions of the tunica media within the bands and extensions of the tunica interna on their luminal surface [2] (Figure 3). The presence of some smooth muscle cells within the bands suggests they may have some degree of contractility [2]. Calcifications have been associated with both BAF and BAB. Small et al. (2021) showed non-atherosclerotic calcification in 5.1 % of the BAF cases and 69.1% of the BAB cases [4]. Atherosclerosis was noted in the anterior and posterior cerebral circulation in cases of patients with BAB [2,4,6]. BAF and intraluminal arterial webs have been associated with plaque formation, transient ischemic attacks, and cerebral infarcts [8-13]. However, no case of thrombus was previously reported. 

Strokes are the second leading cause of death worldwide, and VB stroke is reportedly responsible for up to one-fourth of all strokes and transient ischemic attacks [16,17]. Traditionally, VB strokes have been considered to have more benign outcomes compared to anterior circulation strokes. However, the data conflicts with some studies indicating that VB stroke patients may experience higher levels of impairment [16,18]. Strokes in the posterior circulation are commonly due to thromboses generated from atherosclerotic plaques in the vertebral artery, basilar artery, and proximal posterior cerebral artery [19,20]. Typically, in areas of turbulent blood flow, a thrombus forms over a ruptured plaque or an intact plaque with superficial endothelial erosion. It can be suggested that the presence of BAB creates turbulent and high shear circulation, which can lead to the formation of a thrombus over an atherosclerotic plaque. This could explain the BAB-associated thrombus observed in our case.

## Conclusions

The exact clinical implications of BAB have not been fully elucidated; however, they should be considered in cases of VB strokes and by interventionalists and neurosurgeons when performing procedures in the VB circulation.

## References

[1] Davy J (1839). Of a peculiarity of structure occasionally occurring in the basilar artery of man. Edinb Med Surg J.

[2] Glennon SE, Ram K, Gupta T (2022). Basilar artery bands: anatomic and histologic study with application to coiling and stenting procedures. World Neurosurg.

[3] Hassler O (1965). Intra-arterial bridges in the larger cerebral arteries. Acta Radiol Diagn (Stockh).

[4] Small JE, Macey MB, Wakhloo AK, Sehgal S (2021). CTA evaluation of basilar septations: an entity better characterized as aberrant basilar fenestrations. AJNR Am J Neuroradiol.

[5] Stehbens WE (1975). Flow in experimental models simulating intravascular cords traversing the arterial lumen. Vasc Surg.

[6] Tubbs RS, Shaffer WA, Loukas M, Shoja MM, Harrigan MR, Oakes WJ (2008). Intraluminal septation of the basilar artery: incidence and potential clinical significance. Folia Morphol (Warsz).

[7] Iwanaga J, Courville E, Anand MK (2020). Chordae willisii within the transverse sinus: morphologic study. World Neurosurg.

[8] Coutinho JM, Derkatch S, Potvin AR, Tomlinson G, Casaubon LK, Silver FL, Mandell DM (2017). Correction: Carotid artery web and ischemic stroke: a case-control study. Neurology.

[9] Esenwa C, Labovitz D, Caplan LR (2019). “Basilar web” causing basilar branch infarction. J Stroke Cerebrovasc Dis.

[10] Green DM, Caplan LR (2000). Recurrent basilar branch infarcts due to a protruding basilar artery lesion. Cerebrovasc Dis.

[11] Hu H, Zhang X, Zhao J, Li Y, Zhao Y (2019). Transient ischemic attack and carotid web. AJNR Am J Neuroradiol.

[12] Li J, Chen L (2023). Brainstem infarction due to a basilar arterial web. Radiology.

[13] Liu L, Zhang XB, Lu S, Liu ZJ, Zhu XJ (2019). Plaque distribution of basilar artery fenestration by 3D high-resolution MR vessel wall imaging. Cell Transplant.

[14] Klein GE, Szolar DH, Leber KA, Karaic R, Hausegger KA (1997). Basilar tip aneurysm: endovascular treatment with Guglielmi detachable coils--midterm results. Radiology.

[15] Tateshima S, Murayama Y, Gobin YP, Duckwiler GR, Guglielmi G, Viñuela F (2000). Endovascular treatment of basilar tip aneurysms using Guglielmi detachable coils: anatomic and clinical outcomes in 73 patients from a single institution. Neurosurgery.

[16] Carvalho V, Cruz VT (2020). Clinical presentation of vertebrobasilar stroke. Porto Biomed J.

[17] Go S (2015). Posterior circulation ischemic stroke. Mo Med.

[18] Kim JT, Park MS, Choi KH (2017). Clinical outcomes of posterior versus anterior circulation infarction with low national institutes of health stroke scale scores. Stroke.

[19] Piechowski‐Jóźwiak B, Bogousslavsky J (2008). Chapter 26 Posterior circulation strokes. Handbook of Clinical Neurology.

[20] Kuybu O, Tadi P, Dossani RH (2024). Posterior cerebral artery stroke. https://www.ncbi.nlm.nih.gov/books/NBK532296/.

